# Universal health coverage for undocumented migrants in the WHO European region: a long way to go

**DOI:** 10.1016/j.lanepe.2023.100803

**Published:** 2024-05-28

**Authors:** Kerrie Stevenson, Khatia Antia, Rachel Burns, Davide Mosca, Genevieve Gencianos, Bernd Rechel, Marie Norredam, Michele LeVoy, Karl Blanchet

**Affiliations:** a Institute of Health Informatics, University College London, 222 Euston Road, London, NW1 2DA, United Kingdom; b Faculty of Public Health and Policy, London School of Hygiene & Tropical Medicine, Keppel Street London, WC1E 7HT, United Kingdom; c Heidelberg Institute of Global Health, University Hospital Heidelberg, Im Neuenheimer Feld 130.3, 69120, Heidelberg, Germany; d Realizing SDGs for Migrants, Displaced, and Communities, Italy; e Public Services International, Head Office, France; f European Observatory on Health Systems and Policies, London School of Hygiene & Tropical Medicine, London, United Kingdom; g Department of Public Health, Danish Research Center for Migration, Ethnicity and Health, Section of Health Services, University of Copenhagen, Denmark; h Platform for International Cooperation on Undocumented Migrants (PICUM), Brussels, Belgium; i Geneva Centre of Humanitarian Studies, Faculty of Medicine, University of Geneva, Switzerland; j Lancet Migration European Region Hub, Switzerland

**Keywords:** Public health, Migration, Health policy

## Abstract

The number of people on the move internationally is increasing, and a sizable number of these individuals are migrating through and to the WHO European Region. The UN Sustainable Development Goals demand that we leave no one behind and ensure equitable implementation of Universal Health Coverage (UHC), regardless of immigration status. In the WHO European region, some of the migrants in the most precarious situations are undocumented; defined as those who may have been unsuccessful in asylum applications, born to undocumented parents, continued their residence in a country after their permit or other means of stay expired, as well as those who have entered the country irregularly. These undocumented migrants face some of the biggest challenges to accessing UHC and are often left behind by systems that exclude and stigmatise them. This paper examines the literature on access to healthcare for undocumented migrants in the WHO European Region and calls for urgent action towards ensuring UHC for all migrants regardless of immigration status by 2030.

**Key Recommendations**

▪ Significant action is needed to ensure universal health coverage (UHC) for all people, regardless of immigration status, in line with the sustainable development goals (SDGs). European region governments must extend the legal entitlements to UHC to the entire resident population, including undocumented migrants.
▪ International organisations must establish comparative data monitoring systems across the European region to assess UHC coverage and the costs and benefits of exclusion of undocumented migrants within health systems. These should include disaggregated data on migration status, minority groups, including ethnic minorities, age, and gender.
▪ National policies prioritising social integration, better housing, safe working conditions, and a fair immigration system are crucial to improving health outcomes for undocumented migrants in the European region.
▪ Governments and healthcare institutions must implement firewalls to ensure undocumented migrants can access healthcare without fear that their personal data will be used for immigration enforcement.
▪ Any UHC initiatives must be underpinned by a participatory approach which places undocumented migrants at the centre of both design and implementation.
▪ Practitioners and researchers should ensure better research on the health status of undocumented migrants in the European region, including their determinants of health, particularly in Eastern Europe where data are scarce.

## Introduction

International migration is an important determinant of health and has important implications for the provision of Universal Health Coverage (UHC) in the World Health Organization (WHO) European region. Including migrants in the health system is an essential contribution to their social integration and key to the moral imperative stipulated by the United Nations (UN) in the Sustainable Development Goals (SDGs) of ‘Leaving No One Behind’ and the drive towards UHC by 2030.^1^^,^^2^ UHC is based on the principle that all individuals and communities should have access to quality essential health services without suffering financial hardship, regardless of an individual's immigration status.^3^ In June 2023, the Third Global Consultation on the Health of Refugees and Migrants reaffirmed the right of every human being, without distinction of any kind, to enjoy the highest attainable standard of physical and mental health. Additionally, it called for explicit recognition of undocumented migrants in UHC initiatives, and stressed the inclusion of all migrants in UHC regardless of immigration status.^4^

**Search strategy and selection criteria**

We conducted a rapid review of relevant literature from 2018 to 2023 in preparation for this article. The previous five-year period was chosen to provide the most recent literature in this field, and capture the major shifts associated with the COVID-19 pandemic and the recent migration crises. Our search was primarily conducted through the PubMed database complemented by grey literature. We concentrated on three distinct migrant groups: undocumented migrants; refugees; and asylum seekers and conducted two separate searches using the following keywords: “undocumented migrants AND health access AND Europe” and “refugee OR asylum seeker AND health access AND Europe”. While refugees and asylum seekers are not the central focus of this study, we included them to allow a comparison between the number of published studies, geographic scope, and outcomes. The search retrieved 483 results (178 relating to undocumented migrants and 305 relating to refugees or asylum seekers). After title and abstract screening, 266 articles (78 relating to undocumented migrants and 188 relating to refugees and asylum seekers) were considered potentially relevant. Subsequently, these articles underwent a rigorous full-text screening resulting in 48 articles for inclusion. Literature relating only to refugees and asylum seekers was excluded at the full-text screening stage. We also conducted a search of the reference lists of included articles. We conducted searches on the websites of the following organisations using the same keywords: Migrant Integration Policy Index (MIPEX); Platform for International Cooperation on Undocumented Migrants (PICUM); Universal Health Coverage 2023 Platform; The International Organization for Migration (IOM); and the European Union Agency for Fundamental Rights (FRA). Relevant grey literature was identified through a Google Scholar search, and the same criteria for inclusion were applied. In addition, other relevant articles were included based on the authors’ recommendations.

Despite all UN member states agreeing to the SDGs, including UHC for all, evidence suggests that migrants across Europe often face barriers in accessing health services. Some of the migrants in the most precarious situations are undocumented. Undocumented migrants are defined as persons who do not fulfil the administrative requirements established by the country of destination to enter, stay, or exercise an economic activity.^5^^,^^6^ Undocumented migrants are a heterogenous group, including those who have been unsuccessful in asylum applications (unsuccessful asylum-seekers), children born to undocumented parents, those who have continued their residence in a country after their permit or other means of stay expired, as well as those who have entered the country irregularly.^5^, ^6^, ^7^ Refugees are defined as people who ‘owing to a well-founded fear of being persecuted for reasons of race, religion, nationality, membership of a particular social group, or political opinion, is outside the country of his nationality, and is unable to or, owing to such fear, is unwilling to avail himself of the protection of that country’.^8^^,^^9^ They may be granted a special legal protection status in a host country after going through the asylum system.^8^ Undocumented migrants are sometimes referred to as ‘illegal’, ‘illegalized’, or ‘irregular’, but these terms have been criticised by both the UN and European Union (EU).^10^^,^^11^ Critics argue they deny a person their basic humanity, imply they are criminals who are undeserving of rights, and perpetuate negative public perceptions of undocumented migrants.^11^, ^12^, ^13^

The exact number of undocumented persons currently living in Europe is unknown and existing estimates are contested. In 2008, a European Commission-funded study estimated that 1.9–3.8 million undocumented migrants resided in the EU, accounting for 0.39%–0.77% of the total population.^14^ A newer study funded by the European Commission, due for publication in 2025, is currently working on new estimates of undocumented migrants in various countries of the EU.^15^ Migrants often shift between administrative categories, and undocumented migrants can become asylum seekers or refugees, and vice versa.^5^, ^6^, ^7^ Undocumented migrants are often in the most precarious positions in society and often have insecure housing and employment and poor access to basic services, and experience significant racism and discrimination.^7^^,^^16^ Given their precarious status and lack of legal rights, they are also at higher risk of experiencing human trafficking, modern slavery, and insecure or dangerous employment which has a direct impact on their health and healthcare access.^17^^,^^18^ Indeed, the level of access to healthcare amongst undocumented migrants can be regarded as the ultimate barometer for UHC in the European region, and an indicator of health systems’ inclusiveness.^19^

This paper explores undocumented migrants’ access to UHC in Europe. Drawing on academic and grey literature, we synthesise available evidence on healthcare access and barriers for undocumented migrants in the WHO European Region, identify examples of good practice, and make recommendations on how to improve healthcare access for undocumented migrants across the region.

## What is UHC and how does it relate to undocumented migrants in the European region?

bUHC is defined by the WHO as ‘ensuring that all people have access to promotive, preventive, curative, and rehabilitative health services of quality, when and where they need them, without financial hardship.’^2^ The SDGs aim to ensure UHC worldwide by 2030. Progress is tracked using two indicators: (1) coverage of essential health services (SDG 3.8.1); and (2) catastrophic health spending (SDG 3.8.2).^1^

**Fig. 1:**
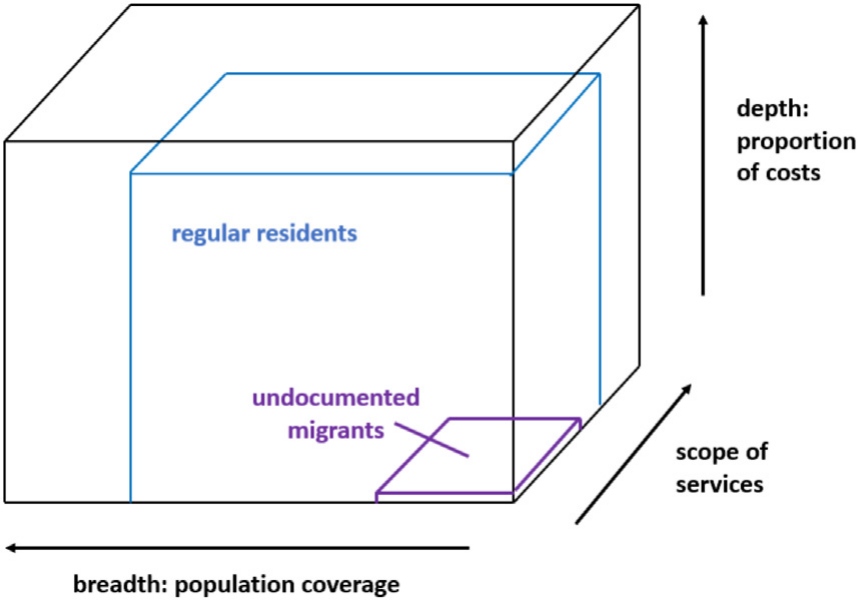
Universal Health Coverage for regular residents and undocumented migrants in Norway.

Source: Authors' compilation, adapted from: https://gh.bmj.com/content/3/5/e001031.^19^

UHC has three dimensions: (1) Population: this relates to who is covered in the general population, the ‘breadth’; (2) Services: this dimension relates to the question of which types of healthcare procedures or medications are covered; (3) Costs: this dimension relates to the proportion of healthcare charges covered, ‘the depth’. In general, due to fiscal constraints on public spending, it is challenging for governments to provide UHC for all types of health services for residents. This can be represented in a ‘UHC Coverage Cube’ (Fig. 1). The publicly paid share of the coverage cube is typically smaller than 100% in all three dimensions, depicted by the blue cube in Fig. 1. However, in many countries coverage is drastically smaller for undocumented migrants, resulting in a much smaller cube; depicted in Fig. 1 as a purple cube.

## What do we know about the health status of undocumented migrants in the European region?

There is a dearth of evidence on undocumented migrants' health needs and determinants of health across the European region.^20^, ^21^, ^22^ A literature search by the authors in the database PubMed identified less than 300 research articles published in English on undocumented migrants’ health in Europe between 2018 and 2023. Conversely, almost 6000 research papers were identified on refugee health issues in Europe. Our exploratory findings are corroborated by a recent scoping review exploring global evidence on mental health services among migrants in primary healthcare settings in Europe; the authors could not identify a single study primarily addressing the undocumented migrant population.^23^ Most research on the health of undocumented migrants in Europe originates from Western European countries including Switzerland, United Kingdom, Germany, Netherlands, Sweden, Finland, and Norway, with almost no studies conducted in Eastern Europe.^20^, ^21^, ^22^^,^^24^^,^^25^

The research that has been undertaken highlights that undocumented migrants are susceptible to chronic physical health conditions and mental health disorders.^21^^,^^24^^,^^26^^,^^27^ Research from Switzerland found that in primary care settings, undocumented migrants presented a variety of health issues and were disproportionately affected by chronic illnesses, with more than 70% of the study sample reporting at least one chronic condition and more.^21^ Post-traumatic stress disorders (PTSD), anxiety, and depression were the most reported mental health outcomes among undocumented migrants in Sweden.^25^ Additionally, given their precarious migration status and susceptibility to exploitation, they are at high risk of interpersonal violence, trafficking, and abuse.^28^^,^^29^ One study in the Netherlands found that more than a third of help-seeking undocumented migrants recorded physical and sexual assault.^30^ Furthermore, given the precarious nature of undocumented migrants’ living conditions and complex migration journeys, those suffering from long-term illnesses lacked comprehensive health records, access to medication, and continuity of care, meaning their longer-term health outcomes are poorer.^29^^,^^31^^,^^32^

Compared to the adult population, the literature on the health of undocumented migrant children is even more limited. Despite the efforts of WHO and its member states to ensure full vaccination coverage in Europe, little is known about the immunisation status of undocumented migrant children. However, small scale studies suggest they are often in need of routine childhood vaccinations on arrival in host countries.^33^, ^34^, ^35^ Children of undocumented migrants in Sweden often remain unvaccinated or without any access to basic health services because parents are afraid of being questioned about their migration status, or they mistrust healthcare providers and prefer to avoid any contact with the health system.^33^ Historical abuses relating to inappropriate administration and testing of medications amongst minority ethnic groups serve to further entrench this mistrust.^36^ Likewise, undocumented pregnant women are at severe risk of poor outcomes. For example, in Finland and Denmark undocumented pregnant women experience long delays to access antenatal care and others never attend antenatal care appointments prior to labour.^37^^,^^38^ Pregnant women living in Finland without regular immigration status have significantly higher rates of infectious illnesses and inadequate prenatal screening coverage, and recent Danish studies have demonstrated higher risk of stillbirth and preterm birth amongst undocumented migrant women.^37^^,^^39^ Evidence also suggests high maternal mortality, severe acute maternal morbidity, preterm birth, and low birth weight among this population across Europe.^40^

## What are the legal policies and frameworks relating to health services for undocumented migrants in the European region?

Legal entitlements to publicly paid health services for undocumented migrants differ widely across the European region, with many countries restricting access even to essential and emergency services.^41^^,^^42^ However, it is challenging to make a comprehensive assessment of entitlements and their implementation, given a lack of data collection and systematic monitoring mechanisms.^34^ One comparatively recent approach is the Migrant Integration Policy Index (MIPEX) which aims to quantify the level of integration of migrants across six continents using various integration policy indicators, including access to healthcare.^43^ The scores are based on the assessment of national experts who suggest a quantitative score according to prespecified policy indicators. The most recent assessment was conducted in 2019, and unsurprisingly undocumented migrants faced the greatest barriers to obtaining UHC coverage. There was little evidence to suggest entitlements for undocumented migrants had improved since the last MIPEX assessment in 2014. MIPEX do not provide disaggregated scores for undocumented migrants, but the written report highlighted that only one European region country–Switzerland–imposed no administrative barriers for undocumented migrants.^43^ In countries where coverage is limited to emergency care for undocumented migrants, a barrier exists in the form of a discretionary judgement about whether the health problem constitutes an emergency.^43^ In 2020, the NowHereLand ‘In Times of Pandemic Project’ similarly found that in most European countries undocumented migrants were not entitled to the full range of publicly paid health services and that they were also denied access to social welfare, compulsory education, housing and work, all of which have negative repercussions on an individual's health status.^44^^,^^45^ A summary of the report's health and social welfare findings for some European region countries are presented in Table 1 (note disaggregated data are not available for all countries). Both MIPEX and the NowHereLand reports highlight that certain health services or health conditions are prioritised more than others. This includes maternal and child health and infectious diseases, for which health services are often offered free of charge, while longer-term chronic conditions and general adult healthcare tend to be excluded.^42^, ^43^, ^44^, ^45^ These international and regional policy analyses provide a helpful overview of access to healthcare and other key services for undocumented migrants, but few present country-specific disaggregated data for undocumented migrants, and are usually based on the opinions of a limited number of policy experts.

**Table 1:** A summary of healthcare and social welfare coverage for undocumented migrants in the European region as presented in the NowHereLand ‘In Times of Pandemic Project’.

The possibility of an alternative approach became clearer during the COVID-19 pandemic, when some European countries, such as Belgium, Greece, Ireland, Portugal, Spain, Turkey and the United Kingdom, extended free access to the COVID-19 vaccination to undocumented migrants.^46^^,^^47^ Some of these countries, such as Portugal, went further and regularised the status of all people with a pending residence application.^48^ This has benefited an estimated 350,000 people in Portugal, and boosted uptake of the COVID-19 vaccine.^48^^,^^49^ Since then, Portugal has continued to offer undocumented migrants the ability to apply for residence, and the right to request family reunification visas, although the waiting time for processing application continues to increase.^49^ As a result of their approach to regularising undocumented migrants, Portugal's labour force has grown and many believe this approach has strengthened the economy.^49^

Another example of how health systems can become more inclusive emerged after Russia's invasion of Ukraine in 2022, when the Temporary Protection Directive 2001/55/EC was activated for the first time in the EU, enabling immediate access to healthcare for Ukrainian citizens fleeing Ukraine and residing in the EU.^50^ The directive exists for ‘mass influx or imminent mass influx of displaced persons from non-EU countries who are unable to return to their country of origin’, but does not apply to most third country nationals and undocumented migrants fleeing Ukraine.^51^^,^^52^ The European Commission also called for urgent access to routine vaccinations for children, as well as targeted action on mental health and trauma. This has been well received across EU countries, and despite the challenges, many thousands of people fleeing Ukraine have been able to access healthcare. Nonetheless, the directive was not applied when many other non-EU migrants reached the region over the past decades whilst fleeing conflict, including people from Syria, Afghanistan, or Iraq.^51^^,^^52^ Some suggest Ukrainian migrants have been viewed more favourably and welcomed by European communities when compared to other migrant groups, with some believing this is due to racism and xenophobia.^53^ The EU-wide response to the war in Ukraine demonstrates that when there is the political willingness to facilitate equitable access for migrants, it is possible. However, it also demonstrates that inclusivity should include all regardless of ethnicity and immigration status.

It is important to also recognize local civil society-led initiatives to improve access to healthcare for undocumented migrants. Where mainstream healthcare services and legal frameworks fail to provide publicly paid health services for undocumented migrants, non-governmental organisations (NGOs) and individuals play a key role in service provision. Their role sometimes begins as soon as migrants arrive in the European region, such as for those who arrive via the Mediterranean Sea. Historically, EU-led Search and Rescue operations offered medical care to newly arrived migrants, but increasingly NGOs are filling the gaps where EU or state funding has been removed.^54^ In 2011, Norway granted undocumented women the right to antenatal care and birth at government hospitals but did not include them in all primary care or reimbursement schemes.^55^ Consequently, NGOs established health clinics for undocumented migrants in Norway's two largest cities.^55^ Likewise, in the UK, Médicins du Monde (MdM) offer a free healthcare clinic in Central London for marginalised people, including undocumented migrants, and support them to access healthcare services and immigration advice.^56^ At present, undocumented migrants must pay 150% of NHS care costs for any maternity care and non-emergency secondary care, and NGOs such as MdM play a key role in ensuring access to mainstream healthcare for this underserved group.^57^ The COVID-19 pandemic demonstrated the importance of NGOs in service provision for undocumented migrants. For example, in Italy, the government funded a number of NGOs to support undocumented migrants with health and social care, including in accessing the COVID-19 vaccination.^58^ Anecdotal reports also suggest there are countless individual clinicians working at the local level throughout the WHO European Region who offer discretionary free or reduced cost care to undocumented migrants. The work of these NGOs and individuals is to be commended; their role is often undervalued and under-recognised. However, although NGO efforts are currently vital, they cannot be regarded as a long-term solution to the problems with access to UHC for undocumented migrants in countries and the region.^41^

## What are other facilitators and barriers to UHC for undocumented migrants in the European region?

Accessing healthcare can be challenging for several reasons not related to existing UHC-related legal instruments. Policies intended to ensure meaningful healthcare access for undocumented migrants are not necessarily implemented as intended, due to a number of factors, including insufficient funding, discrimination within the health system, and poor monitoring and training of health workforce.^28^ This is sometimes referred to as the ‘implementation gap’.^59^

Other challenges faced by migrants include fears of discrimination or racism, language barriers and lack of interpretation services, concerns about data-sharing with immigration authorities, and high out-of-pocket healthcare costs.^28^ Populism is a major challenge in many European countries, leading to anti-immigration sentiment and restrictive migration policies.^60^ Increasingly, undocumented migrants are not viewed as valid members of society, but are ‘othered’, cast as the stranger, and often denied the hospitality traditionally shown to strangers.^61^ Many countries view undocumented migrants as a threat and focus predominantly on deterring entrance and increased costs instead of the economic and cultural benefits of migration.^60^ In addition, many European countries are actively seeking to deter undocumented migrants from entering through physical blockades, military patrols, and harsher laws; so-called ‘Fortress Europe’.^62^ For example, the UK government recently agreed a plan to send asylum seekers to Rwanda for asylum processing claiming this was a more cost-effective way of managing their asylum backlog and deterring further asylum applicants.^63^^,^^64^ In this context, healthcare workers may be unwilling to offer care to undocumented migrants, for fear of reprisal from authorities or lack of awareness of entitlements.^28^ This is worsened by the lack of coherence between health and migration policies; the first being tendentially inclusivist (e.g. ‘health for all’) and the second being naturally exclusivist and focused on restrictions and security. Some argue that obstacles to inclusion are generated by financial constraints and austerity, although others argue that financial issues might be a false problem.^65^^,^^66^ For example, the Spanish government removed healthcare coverage for undocumented migrants in 2012, ostensibly in reaction to the financial crisis of 2008.^67^ Similarly, the UK limits access to free healthcare for undocumented migrants, and claims this is to limit national healthcare spending.^68^ However, research suggests that limiting access to free healthcare for undocumented migrants may not be economically beneficial.^65^^,^^69^ For example, some argue extending coverage to undocumented migrants curbs the spread of infectious disease such as tuberculosis (TB) and measles in the general population, thereby reducing overall healthcare spending.^65^^,^^69^ Many countries across the European region offer free emergency care for undocumented migrants, but limit access to other forms of care.^43^^,^^45^ However, emergency care is costly, and data suggest that if primary and preventative care was offered free of charge, it would prevent multiple visits to the emergency department and reduce spending on costly emergency care.^45^^,^^65^ Data also suggest healthcare costs for undocumented migrants are less than those of the general population given undocumented migrants are often younger and more healthy than the general population.^45^^,^^65^ Some also suggest that access to publicly funded healthcare creates a ‘pull factor’ for undocumented migrants, i.e. a country may experience an increase in arrivals from undocumented migrants if they expand healthcare coverage.^70^ However, research has demonstrated that migrants do not usually choose the destination country based on healthcare access.^70^, ^71^, ^72^ Indeed, research has suggested that migration can raise economic prospects in host countries, including in extending permanent residence status to undocumented migrants.^73^, ^74^, ^75^ A 2020 International Monetary Fund (IMF) report found that migration generally improves economic growth and productivity in host countries, particularly in advanced economies where migration increases output and productivity in the short- and medium-term.^73^

The issues surrounding data-sharing between healthcare and immigration authorities can have a severe impact on undocumented migrants' ability to trust the health system, their ability to access the care they are entitled to, and on the availability of healthcare data for undocumented migrants.^76^ For example, in the UK, the government mandated data-sharing between the health system and the Home Office resulted in the deportation of undocumented migrants, and even though this has now stopped, there remains a deep mistrust of the system amongst undocumented migrants in the country.^57^^,^^77^ Even countries with relatively inclusive healthcare policies, many still share data with immigration enforcement teams, which can result in detention or deportation, and thus the benefit of equal access to healthcare may become a barrier. In response to this inequity, the Council of Europe's European Commission Against Racism (ECRI) issued high-level guidelines concerning the creation of effective measures (‘firewalls’) to prohibit social services providers from sharing the personal data of suspected irregular migrants with immigration authorities.^27^ These are the most systematic and comprehensive guidelines to date and make a powerful statement to the global community. The global trade union federation Public Services International (PSI) has also made policy recommendations calling for a firewall between healthcare and immigration enforcement.^78^^,^^79^ Firewalls separate the provision of fundamental social rights and immigration enforcement. They serve to ensure that no data gathered to protect fundamental social rights is shared for immigration control purposes.^80^ Firewalls are key to ensuring we have access to improved data on the healthcare status of undocumented migrants; if undocumented migrants can be assured their information won't be shared with immigration officials it is likely to increase use of healthcare services and thus contribute to improved data collection for this group.

Healthcare workers can act as gatekeepers to equitable implementation of UHC. Thus, it is vital that they have sufficient training to ensure that undocumented migrants are not discriminated against when seeking healthcare.^28^ The WHO have developed global competency standards for health workers in promoting migrant and refugee health that can be implemented across the WHO European Region.^81^ Anti-discrimination policies grounded on human rights, including the right to health, must be integrated in health systems and such information be made available to everyone.^28^^,^^42^ Lack of understanding thereof can lead to discriminatory practices that stigmatize migrants. For example, a study conducted in Sweden demonstrated that some undocumented migrants in maternity services were treated according to ‘deservingness’, with pregnant undocumented migrants finding that they were denied care because they did not pay formal taxes or did not have a settled immigration status, despite being eligible for free maternity care.^82^ The study found that healthcare providers made decisions about deservingness based on ethnicity, residency status, and on ability to speak English.^82^ Holmes et al. describe ‘deservingness’ in the context of migration as a ‘critical awareness of the often unspoken presumptions of which categories of patients are more or less deserving of access to and quality of care, regardless of their formal legal eligibility.’ However, the authors of this paper and many studies also recognise that countless healthcare workers globally show immense kindness to undocumented migrants and offer care regardless of immigration status.^61^^,^^79^^,^^82^

In countries where undocumented migrants have legal entitlements to health care, access may depend on the availability and quality of interpretation services.^28^^,^^83^ Research suggests that access to interpreters is often limited in rural areas or in countries where there are few migrants.^83^ Outreach services have become important entry points into the health system for undocumented migrants in several countries.^84^ Successful outreach services operate within conveniently located, trusted community spaces, and include target communities in designing, delivering, and assessing their services.^84^ A good example of this is an outreach clinic first established in Denmark in 2006.^85^ The ‘AmiAmi’ clinic offers care to all undocumented migrants, but focuses on those who are victims of trafficking and who work as sex workers. The clinic has demonstrated effectiveness in engaging with these underserved migrant groups and demonstrates that a community-based and culturally appropriate service can improve access to UHC for undocumented migrants.

It is also important that undocumented migrants are equipped with knowledge about the health system and their entitlements to publicly paid services. Where this aspect of health literacy is underdeveloped, underutilisation of services and delays in accessing urgent and essential care follow.^28^ This also applies to health workers, so that they do not engage in discriminatory behaviour or the ad-hoc application of regulations.^28^ For example, research conducted in the UK revealed that healthcare professionals lack confidence in comprehending and implementing the charging regulations for undocumented children's health services.^86^ A study from Spain suggested that undocumented migrants have difficulties accessing health services because of ongoing systemic constraints, frequent and ambiguous rule changes, and the necessity to negotiate discrepancies between autonomous communities within the country.^87^ Programmes that support understanding of rights and entitlements and health literacy more generally for undocumented migrants should be co-designed with communities and delivered using culturally and linguistically appropriate methods.^88^ It is vital that such efforts seek to address the power imbalances present between healthcare professionals and undocumented migrants.^89^ The benefits of a co-produced approach include ensuring outputs are directly relevant to undocumented migrant communities, and that any healthcare initiatives consider the wider determinants of health, which are often key priorities of underserved migrant communities. Additionally, both research and action to solve this crisis must apply intersectional thinking and interdisciplinary work to maximise effectiveness across all sectors.

## Conclusion—a call to action

This article has highlighted some of the healthcare inequities faced by undocumented migrants in the European region, and examples of where governments and NGOs have taken action to bridge the gaps in healthcare access. Significant action is needed to ensure UHC for all people, regardless of immigration status, in line with the SDGs.

This is primarily the task of national governments, all of which have signed up to the SDGs and international legal instruments, including those that recognise the right of all to the highest attainable level of physical and mental health. To achieve these international goals, governments must urgently and explicitly extend the legal entitlements to UHC to the entire resident population, including undocumented migrants. However, this is only the first step. As illustrated by the UHC cube, entitlements have two more dimensions—the scope of publicly paid services and the share of costs that is publicly covered. In both dimensions, undocumented migrants must be on par with the rest of the population. In addition to the lack of legal entitlements to health care in many countries, undocumented migrants face additional barriers to accessing services including the fear of deportation, racism, as well as the lack of interpreting services and lack of culturally appropriate care. If undocumented migrants should have the same access to health services as the rest of the population, these barriers must be overcome. The approach to the Ukrainian war has demonstrated that governments can take swift action to integrate migrants into health systems, but this must include all migrants regardless of immigration status, ethnicity, or nationality. Governments and healthcare institutions must urgently implement firewall policies enabling undocumented migrants to safely access healthcare without fear that their personal data will be used for immigration enforcement. In addition, national policies that encourage social integration, better housing, safe working conditions, and a fair immigration system are crucial to improving health outcomes.

NGOs, healthcare organisations, practitioners, and researchers must work to ensure courageous translation of knowledge into action on healthcare equity for undocumented migrants. Any initiatives must be underpinned by a participatory approach which places undocumented migrants at the centre of both design and implementation. Given the high burden of multimorbid and chronic diseases among undocumented migrants, healthcare professionals and NGOs must provide comprehensive and inclusive healthcare for undocumented migrants, especially where governments have failed to do so. We also call upon practitioners and researchers to ensure better research on the health status of undocumented migrants in the European region, including their determinants of health, particularly in Eastern Europe where data are scarce.

Finally, we call upon international organisations to establish standardised and comparative data monitoring systems across the European region to assess UHC coverage and the costs and benefits of exclusion of undocumented migrants within health systems. These should include disaggregated data on migration status, minority groups, including ethnic minorities, age, and gender. International processes including the 2023 UN General Assembly (UNGA) SDG Summit, the Pandemic Prevention, Preparedness and Response (PPPR) Meeting, and the 2023 UN High-level meeting on UHC should explicitly recognise undocumented migrants as high-risk groups in all recommendations.

There are only a few years left to reach the 2030 SDGs and achieve UHC for all. These may seem to some to be lofty international goals with no real relevance to their countries, but how people living in the most precarious situations, including undocumented migrants, are treated, speaks volumes about the dignity and human rights afforded to all. There is no better time to safeguard the right to health for undocumented migrants in Europe than now.

## Contributors

KS, KB, RB, KA conceived of the article. KS, KB, RB, KA, DM, GC, BR, MN, ML developed the approach. KA developed the search strategy. KS, RB, BR, and KA wrote the first draft, and all authors were invited to comment. KS is the guarantor of the review. All authors approved the final version of the paper.

## Declaration of interests

All opinions expressed are the views of the individual authors and not their employing organisations. The authors declare no conflicts of interest.
